# 25-Gauge Vitrectomy in Open Eye Injury with Retained Foreign Body

**DOI:** 10.1155/2017/3161680

**Published:** 2017-01-09

**Authors:** G. Sborgia, N. Recchimurzo, A. Niro, L. Sborgia, A. Sborgia, G. Alessio

**Affiliations:** ^1^Department of Medical Basic Sciences, Neuroscience and Sense Organs, University of Bari “A. Moro”, Bari, Italy; ^2^Eye Clinic, Azienda Ospedaliero Universitaria Policlinico di Bari, Piazza Giulio Cesare, No. 11, 70124 Bari, Italy

## Abstract

*Purpose*. Ocular trauma with retained foreign body is an important cause of visual impairment in working-age population. Clinical status impacts on the timing and planning of surgery. In the last year small gauge vitrectomy has become safer and more efficient, extending the range of pathologies successfully treated.* Aims*. To evaluate the safety and outcomes in patients with open eye injury with retained foreign body that underwent early 25-gauge vitrectomy.* Methods*. In this retrospective, noncomparative, interventional case series, we performed 25-gauge vitrectomy on 10 patients affected by open globe injuries with retained foreign body, over 3 years. We analyzed age, wound site, foreign body characteristics, ocular lesions correlated, relative afferent pupillary defect, visual acuity, and intraocular pressure. Follow-up evaluations were performed at 1, 3, and 6 months. According to the clinical status we performed other procedures to manage ocular correlated lesions.* Results*. The median age of patients was 37 years. The foreign body median size was 3.5 mm (size range, 1 to 10 mm). 25-gauge vitrectomy was performed within 12 hours of trauma. Foreign body removal occurred via a clear corneal or scleral tunnel incision or linear pars plana scleral access. Visual acuity improved in all patients. Endophthalmitis was never reported. Only two cases reported postoperative ocular hypertension resolved within the follow-up. Retinal detachment recurred in one case only.* Conclusions*. 25-gauge vitrectomy could be considered as early approach to manage open globe injuries with a retained posterior segment foreign body in selected cases with good outcomes and low complication rate.

## 1. Introduction

Ocular trauma with intraocular foreign bodies (IOFBs) is an important cause of visual morbidity and blindness in working-age population [1–3]. The role of vitrectomy as early approach to ocular eye injuries with IOFBs was widely supported by the literature [4–6]. The advancement in microsurgical vitreoretinal surgery techniques and instrumentation has allowed managing successfully traumatized eyes with IOFBs [7, 8]. In very few reports 25-gauge vitrectomy surgery was successfully used for the removal of foreign bodies and to manage ocular lesions using different maneuvers [9–11]. In this case series we report our experience in treating open eye injuries with IOFBs by 25-gauge pars plana vitrectomy in order to evaluate the final visual acuity, globe survival, and complication rate and to describe the proceedings of IOFBs removal and the management of ocular lesions correlated.

## 2. Methods

The setting was the Department of Ophthalmology, University of Bari, Bari, Italy. Over 3 years (2013–2015) ten consecutive patients affected by ocular trauma with retained IOFBs were included in this retrospective, noncomparative, interventional case series. At presentation we analyzed relative afferent pupillary defect (RAPD), Snellen best corrected visual acuity (BCVA), anterior segment by slit lamp biomicroscopy, intraocular pressure (IOP), and posterior segment by funduscopy. Ancillary tests like B-mode ultrasonography and computed tomography (CT) were performed to analyze ocular and orbital status and to detect the localization of the IOFBs. All patients were treated by 25-gauge pars plana vitrectomy by a single physician. According to the clinical status we performed other procedures like lens extraction (lensectomy via pars plana or phacoemulsification and aspiration through a corneal incision), sulcus or capsular bag intraocular lens (IOL) implantation, and repair of retinal break or detachment. A long-term ocular endotamponade was used when necessary. Postoperatively all patients received antibiotics and steroid eye drops for four weeks with gradual tapering. Oral ciprofloxacin 500 mg twice daily was given in all cases with addition of systemic steroids when necessary.

We analyzed age, wound site, IOFB characteristics (chemical nature, size, and location), ocular lesions correlated, site and method for extraction of foreign bodies, and timing of surgery. Intraoperative and postoperative complications were recorded. Follow-up evaluations were performed at 1, 3, and 6 months.

## 3. Surgical Approach

At first the entrance wound was cleaned of any incarcerated tissues and incarcerated vitreous was cut as near to the wound as possible. The scleral, limbal, or corneal entrance wound was repaired before trocars were positioned.

Afterwards pars plana vitreous surgery was performed. When choroidal detachment occurred, use of a 6-mm length infusion cannula was considered.

According to the clinical status we performed other procedures to manage ocular lesions correlated. Four patients underwent small incision phacoemulsification for traumatic cataract at the same time of the vitrectomy. Four patients underwent lensectomy by 25-gauge vitrector handpiece. In a 40-year-old man we removed his relatively soft lens dislocated in the vitreous cavity by 25-gauge vitrector handpiece. In three patients we realized primary sulcus IOL implantation. Six eyes were left aphakic in order to place IOL after an improvement of ocular conditions and an accurate calculation of the IOL power. Core vitrectomy was performed before identifying retained foreign body. Active bleeding was controlled by elevating the infusion or perfluorocarbon liquid or endodiathermy.

In all patients meticulous removal of the vitreous was performed at vitreous base and around the impact site, and posterior vitreous detachment (PVD) was induced.

**Figure 1:**
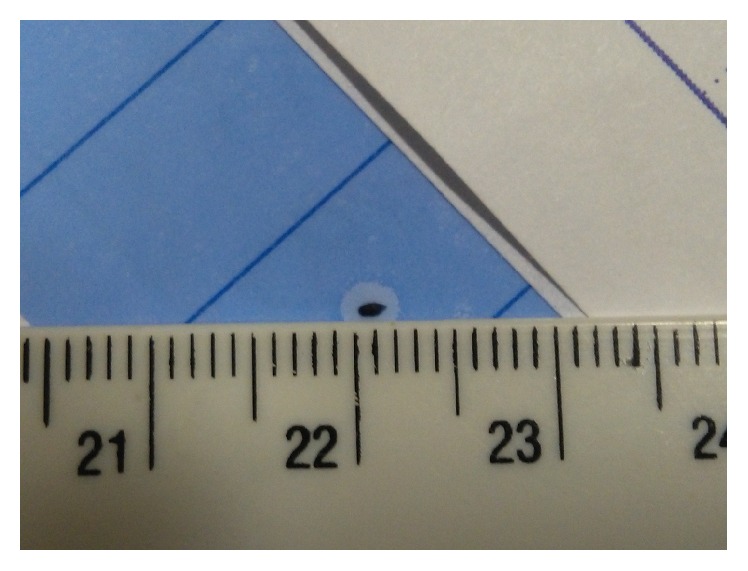
Small metallic IOFB.

Clinic evaluation, ultrasonography, and CT helped us to plan the way of retained foreign body removal. In six patients removal of foreign bodies with small to medium size (range size, 1 to 4 mm) and a regular contour (Figure 1) occurred via a clear corneal or scleral tunnel incision.

**Figure 2:**
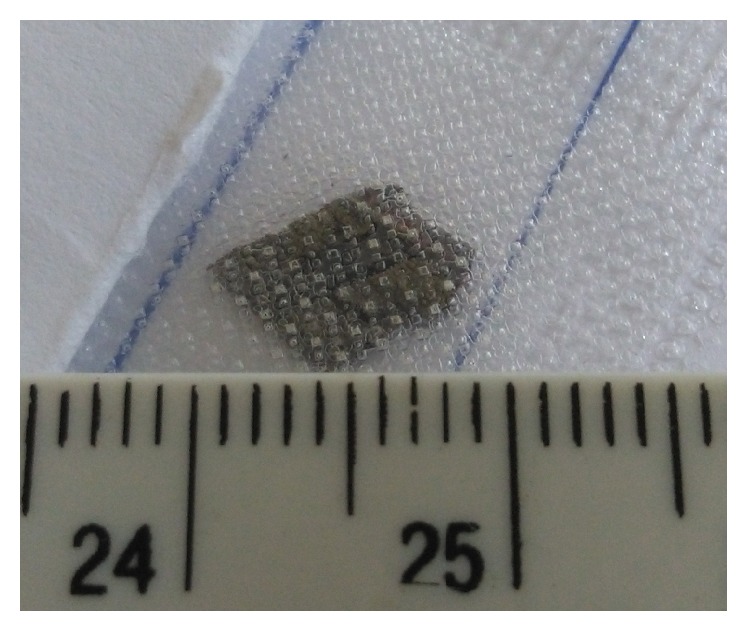
Large stone IOFB.

**Figure 3:**
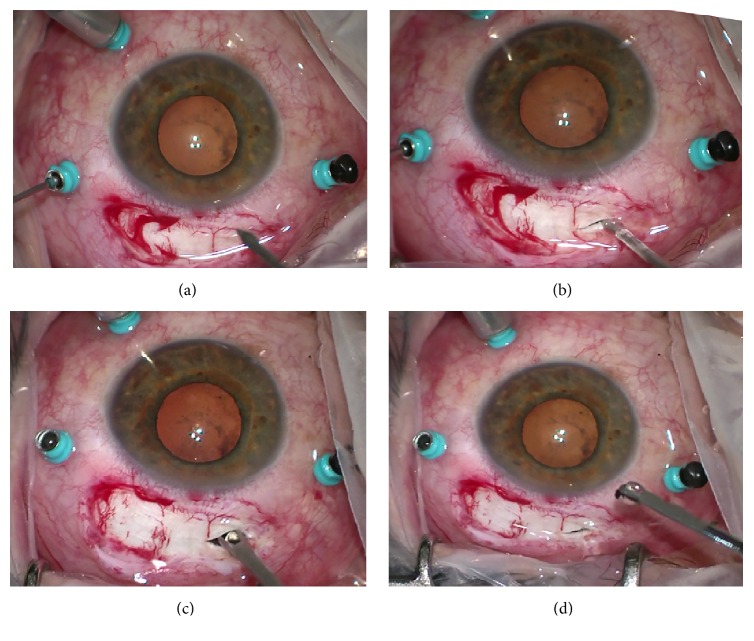
Intraoperative photographs (inverted image as seen by the surgeon). (a-b) Linear pars plana scleral access with max length of 3 mm is realized at 12 hours preserving the sites of trocars. (c-d) A metallic foreign body is extracted through a pars plana sclerotomy by basket forceps to prevent slippage.

In four patients we removed foreign bodies with small to large size (range size, 1,5 to 10 mm) (Figure 2) via linear pars plana scleral access with max length of 3 mm (surgeon decided on appropriate size to ensure safety space for the IOFB removal) realized at 12 hours (Figure 3). For large and more posteriorly located foreign body we used perfluorocarbon liquids to protect the macula against IOFB during its removal. All IOFBs were removed from the posterior segment using intraocular forceps and a retractable basket.

Retinal tears and rhegmatogenous retinal detachment were treated. The periphery was evaluated at the end of surgery meticulously.

In six patients medium viscosity silicone oil (1000 centistokes) was injected with a mean time of 4 ± 2 months between injection and removal; the remaining patients underwent air tamponade. For endophthalmitis prophylaxis, intravitreal injection of vancomycin (1.0 mg/0.1 mL) and ceftazidime (2.25 mg/0.1 mL) was performed. In patients with penicillin allergies, intravitreal amikacin (0.4 mg/0.1 mL) or moxifloxacin (100 *μ*g/0.1 mL) was used.

## 4. Results

We treated 10 patients affected by ocular trauma with retained foreign bodies. The median age was 37 years (range, 23 to 64 years). The entrance wound involved cornea in four cases, sclera in two cases, and limbus in four cases. At presentation six cases showed uveal prolapse, three of those with vitreous incarceration in the wound. Seven patients had traumatic cataract, one patient had lens subluxation, and another one had lens dislocation into the vitreous. In one patient there was no lens displacement.

B-scan ultrasonography revealed or confirmed, after funduscopy, vitreous hemorrhage in seven patients, retinal detachment in seven patients, and choroidal hemorrhage in five patients.

CT localized metallic IOFBs in six cases, a wooden IOFB in one case, a glass IOFB in one case, and a stone in one case but failure to detect intraocular plastic fragment resulted in one case. The IOFBs median size was 3 mm (size range, 1 to 10 mm).

In two patients RAPD was reported while it was not evaluable owing to anterior segment status in three patients. The mean preoperative BCVA was 2 logMAR (Snellen Equivalent (SE) 20/2,000). The mean postoperative BCVA improved to 1.3 logMAR (SE 20/400), 0.95 logMAR (SE 20/178), and 0.85 logMAR (SE 20/142), at 1, 3, and 6 months, respectively. We did not consider the three patients with light perception to quantify mean visual acuity before surgery. In one of those patients visual acuity improved to 0.5 logMAR (SE 20/63) after 6 months from surgery.

At presentation the mean preoperative IOP was 9.4 ± 5.3 mmHg and four patients had hypotony (5 mmHg). Mean postoperative day 1 IOP was 13.6 ± 4.9 mmHg. Mean IOP was 14.8 ± 4.4 mmHg, 14,3 ± 4.1 mmHg, and 15 ± 5.6 mmHg at 1, 3, and 6 months after surgery, respectively. In one patient we recorded hypotony (7 mmHg) resolved spontaneously and in another one hypertony (30 mmHg) successfully managed by eye drops. 25-gauge vitrectomy was performed within a mean time of 12 hours from trauma (time range, 6 to 36 hours). At presentation and follow-up visits endophthalmitis was not reported. Retinal detachment recurred in one patient only and did not occur in the cases without retinal detachment at presentation.

## 5. Discussion

Pars plana vitrectomy is considered the most effective and safest approach for the removal of retained ocular foreign bodies and repair of retinal injuries correlated [12–15].

Advances in small-gauge (25-gauge or 27-gauge) vitrectomy instrumentation as well as surgical techniques have increased indications for complex cases.

First of all small-gauge vitrectomy should allow the best visualization of the intraocular lesions otherwise not detectable permitting the use of wide-angle binocular viewing system with chandelier xenon light source. Poor visualization may influence the efficacy of surgery increasing the risk of iatrogenic lesions, uncompleted removal of IOFB, and insufficient management of ocular injuries correlated, but the delay until the media clear up decreases the chances for vision recovery increasing the risk of complications as inflammatory reaction, proliferative vitreoretinopathy (PVR), endophthalmitis, and toxic reaction. However vitrectomy would be easier waiting for a reduction of corneal edema, traumatic hyphema or fibrinoid reaction, and the spontaneous separation of the posterior vitreous. Many other variables affect the algorithm for IOFB extraction like general medical status, the nature of the trauma, and the chemical nature of the IOFB [16].

If some retrospective analyses claim that the removal within 24 hours reduces incidence of endophthalmitis [17–19] and affects positively the final vision outcome [20, 21], other papers infer that the timing of IOFB removal is not a significant prognostic factor [6, 14]. However these studies have not sufficient power as prospective or randomized clinical trials.

If endophthalmitis is associated with ocular trauma, surgery is urgent. However we schedule a single time approach in order to reduce the risk of endophthalmitis and toxic reactions related to the chemical nature of the IOFB. Noninfectious toxicity is usually associated with metallic IOFBs. More frequently metal is reported in the cases of IOFBs (60% to 88% of IOFBs) [22, 23] as in our case series. Siderosis and chalcosis produce severe inflammation and sterile endophthalmitis hard to manage, so the timing of IOFB removal is an important risk factor for clinical outcome.

The severity of lesions correlated to trauma as scleral wound, vitreous hemorrhage, retinal detachment, also described in our case series, and the time delay of vitrectomy increase the risk of PVR [24]. This knowledge reinforces the belief that the timing of surgery should not be much delayed. After trauma it could be easier waiting at least 2 weeks for a posterior vitreous detachment before performing a vitrectomy but it is not always possible for the ocular lesions correlated and the young age of patients whose vitreous is tenaciously adherent to the retina. Vitrectomy removing the damaged vitreous decreases the risk of retinal detachment. So, using 25-gauge vitrectomy system equipped with a very high cut rates with a preserved duty cycle, surgeon was able to attach vitreous more safely performing a peripheral vitrectomy over detached retina and treating dense hemorrhage or vitreous debris, thanks also to a greater stability of the fluidics. Furthermore a chandelier light source allowed a bimanual technique to approach extreme anterior retina. In eyes with choroidal hemorrhage, confirmed by preoperative ultrasonography, surgeons choose a safer site with a relatively clearer periphery for the placement of trocars and selected a longer infusion cannula to avoid a slippage into the suprachoroidal space.

All the IOFBs were removed from the posterior segment by intraocular forceps with different design to resolve vitreous or fibrin adhesions around the foreign body and basket forceps to prevent slippage of the IOFB and protect scleral access borders. The use of 25-gauge vitrectomy to remove foreign body has also been reported, although an enlargement of the sclerotomy was required in all cases [11]. In this present study, in some cases for the removal of IOFBs we decided to perform scleral access at pars plana to preserve the sites of trocars in order to continue vitrectomy. We did not enlarge sclerotomy to avoid leakage during the later steps of vitrectomy. This technique was employed if the crystalline lens was intact, if the capsule was useful to IOL implant, and it worked for small to very large IOFB having a regular or irregular contour. If lensectomy was performed at the time of vitrectomy and sulcus implant was scheduled, IOFB was gently removed via a clear cornea or scleral tunnel incision. In some cases we combined microincision cataract surgery and 25-gauge vitrectomy without complications and without requiring suturing.

In all patients BCVA improved and also in two patients with RAPD at presentation. Sometimes RAPD evaluation was difficult or even impossible in cases with uveal (iris) prolapse through a corneal or corneoscleral wound.

25-gauge approach generally allows a less traumatic appearance, less conjunctival damage, less intraocular inflammation, and more rapid healing of sclerotomies when compared with 20-gauge PPV [25], although these advantages could seem irrelevant in patients with severe ocular trauma. Different studies have reported no significant difference in endophthalmitis rates between 20 and 25 gauges [26, 27] thanks to improvement in wound making and trocar/cannula entry systems used. Furthermore the design of small gauge and the higher cut rates can ensure a complete vitrectomy reducing the chances of iatrogenic retinal breaks. In our case series the absence of endophthalmitis and the low complication rate about postoperative retinal detachment and IOP alterations comfort us on the safety of our approach although this study included a relatively few patients and literature had reported a variable rate of endophthalmitis [18, 28–30] and postoperative retinal detachment [14, 31, 32] correlated to ocular trauma.

The major limitations of the present study include its noncomparative, retrospective nature. Nonetheless, this present series establishes that sutureless 25-gauge pars plana vitrectomy can be considered a safe and efficacious approach to manage posterior segment IOFBs.

## References

[1] Pieramici D. J., MacCumber M. W., Humayun M. U., Marsh M. J., de Juan E. (1996). Open-globe injury. Update on types of injuries and visual results. *Ophthalmology*.

[2] Entezari M., Rabei H. M., Badalabadi M. M., Mohebbi M. (2006). Visual outcome and ocular survival in open-globe injuries. *Injury*.

[3] Ehlers J. P., Kunimoto D. Y., Ittoop S., Maguire J. I., Ho A. C., Regillo C. D. (2008). Metallic intraocular foreign bodies: characteristics, interventions, and prognostic factors for visual outcome and globe survival. *American Journal of Ophthalmology*.

[4] Demircan N., Soylu M., Yagmur M., Akkaya H., Ozcan A. A., Varinli I. (2005). Pars plana vitrectomy in ocular injury with intraocular foreign body. *Journal of Trauma—Injury, Infection and Critical Care*.

[5] Wani V. B., Al-Ajmi M., Thalib L. (2003). Vitrectomy for posterior segment intraocular foreign bodies: visual results and prognostic factors. *Retina*.

[6] El-Asrar A. M. A., Al-Amro S. A., Khan N., Kangave D. (2000). Visual outcome and prognostic factors after vitrectomy for posterior segment foreign bodies. *European Journal of Ophthalmology*.

[7] Yuksel K., Celik U., Alagoz C., Dundar H., Celik B., Yazlcl A. T. (2015). 23 Gauge pars plana vitrectomy for the removal of retained intraocular foreign bodies. *BMC Ophthalmology*.

[8] Singh R., Bhalekar S., Dogra M. R., Gupta A. (2014). 23-Gauge vitrectomy with intraocular foreign body removal via the limbus: an alternative approach for select cases. *Indian Journal of Ophthalmology*.

[9] Nicoară S. D., Irimescu I., Călinici T., Cristian C. (2015). Intraocular foreign bodies extracted by pars plana vitrectomy: clinical characteristics, management, outcomes and prognostic factors. *BMC Ophthalmology*.

[10] Kunikata H., Uematsu M., Nakazawa T., Fuse N. (2011). Successful removal of large intraocular foreign body by 25-gauge microincision vitrectomy surgery. *Journal of Ophthalmology*.

[11] Kiss S., Vavvas D. (2008). 25-Gauge transconjunctival sutureless pars plana vitrectomy for the removal of retained lens fragments and intraocular foreign bodies. *Retina*.

[12] Mester V., Kuhn F. (1998). Ferrous intraocular foreign bodies retained in the posterior segment: management options and results. *International Ophthalmology*.

[13] Chow D. R., Garretson B. R., Kuczynski B. (2000). External versus internal approach to the removal of metallic intraocular foreign bodies. *Retina*.

[14] Wickham L., Xing W., Bunce C., Sullivan P. (2006). Outcomes of surgery for posterior segment intraocular foreign bodies—a retrospective review of 17 years of clinical experience. *Graefe's Archive for Clinical and Experimental Ophthalmology*.

[15] Yeh S., Colyer M. H., Weichel E. D. (2008). Current trends in the management of intraocular foreign bodies. *Current Opinion in Ophthalmology*.

[16] Mittra R. A., Mieler W. F. (1999). Controversies in the management of open-globe injuries involving the posterior segment. *Survey of Ophthalmology*.

[17] Thach A. B., Ward T. P., Dick J. S. B. (2005). Intraocular foreign body injuries during operation Iraqi freedom. *Ophthalmology*.

[18] Colyer M. H., Weber E. D., Weichel E. D. (2007). Delayed intraocular foreign body removal without endophthalmitis during operations Iraqi freedom and enduring freedom. *Ophthalmology*.

[19] Jonas J. B., Knorr H. L. J., Budde W. M. (2000). Prognostic factors in ocular injuries caused by intraocular or retrobulbar foreign bodies. *Ophthalmology*.

[20] Issac D. L., Ghanem V. C., Nascimento M. A. (2003). Prognostic factors in open globe injuries. *Ophthalmologica*.

[21] Chaudhry I. A., Shamsi F. A., Al-Harthi E., Al-Theeb A., Elzaridi E., Riley F. C. (2008). Incidence and visual outcome of endophthalmitis associated with intraocular foreign bodies. *Graefe's Archive for Clinical and Experimental Ophthalmology*.

[22] Lit E. S., Young L. H. Y. (2002). Anterior and posterior segment intraocular foreign bodies. *International Ophthalmology Clinics*.

[23] Parke D. W., Pathengay A., Flynn H. W., Albini T., Schwartz S. G. (2012). Risk factors for endophthalmitis and retinal detachment with retained intraocular foreign bodies. *Journal of Ophthalmology*.

[24] Feng K., Hu Y., Wang C. (2013). Risk factors, anatomical, and visual outcomes of injured eyes with proliferative vitreoretinopathy: eye injury vitrectomy study. *Retina*.

[25] Kellner L., Wimpissinger B., Stolba U., Brannath W., Binder S. (2007). 25-Gauge vs 20-gauge system for pars plana vitrectomy: a prospective randomised clinical trial. *British Journal of Ophthalmology*.

[26] Park J. C., Ramasamy B., Shaw S., Prasad S., Ling R. H. L. (2014). A prospective and nationwide study investigating endophthalmitis following pars plana vitrectomy: incidence and risk factors. *British Journal of Ophthalmology*.

[27] Scott I. U., Flynn H. W., Acar N. (2011). Incidence of endophthalmitis after 20-gauge vs 23-gauge vs 25-gauge pars plana vitrectomy. *Graefe's Archive for Clinical and Experimental Ophthalmology*.

[28] Schrader W. F. (2004). Epidemiology of open globe injuries: analysis of 1026 injuries in 18 years. *Klinische Monatsblatter fur Augenheilkunde*.

[29] Yang C.-S., Lu C.-K., Lee F.-L., Hsu W.-M., Lee Y.-F., Lee S.-M. (2010). Treatment and outcome of traumatic endophthalmitis in open globe injury with retained intraocular foreign body. *Ophthalmologica*.

[30] Zhang Y., Zhang M. N., Jiang C. H., Yao Y., Zhang K. (2010). Endophthalmitis following open globe injury. *British Journal of Ophthalmology*.

[31] Chiquet C., Gain P., Zech J.-C., Adeleine P., Denis P. (2002). Risk factors for retinal detachment after extraction of intraocular foreign bodies. *Canadian Journal of Ophthalmology*.

[32] Soheilian M., Feghi M., Yazdani S. (2005). Surgical management of non-metallic and non-magnetic metallic intraocular foreign bodies. *Ophthalmic Surgery Lasers and Imaging*.

